# Rigid Polyurethane Biofoams Filled with Pine Seed Shell and Yerba Mate Wastes

**DOI:** 10.3390/polym15092194

**Published:** 2023-05-05

**Authors:** Andrey Pereira Acosta, Agnė Kairytė, Sylwia Członka, Karolina Miedzińska, Arthur Behenck Aramburu, Kelvin Techera Barbosa, Sandro Campos Amico, Rafael de Avila Delucis

**Affiliations:** 1 Postgraduate Program in Mining, Metallurgical and Materials Engineering, Federal University of Rio Grande do Sul, Porto Alegre 91501-970, Brazil; andrey.acosta@ufrgs.br (A.P.A.); arthuraramburu@gmail.com (A.B.A.); kelvintecherabarbosa@gmail.com (K.T.B.); amico@ufrgs.br (S.C.A.); 2 Laboratory of Thermal Insulating Materials and Acoustics, Institute of Building Materials, Faculty of Civil Engineering, Vilnius Gediminas Technical University, Linkmenų St. 28, 08217 Vilnius, Lithuania; 3 Institute of Polymer and Dye Technology, Faculty of Chemistry, Lodz University of Technology, Stefanowskiego 12/16, 90-924 Lodz, Poland; sylwia.czlonka@dokt.p.lodz.pl (S.C.); karolina.miedzinska@dokt.p.lodz.pl (K.M.); 4 Postgraduate Program in Materials Science and Engineering (PPGCEM), Technology Development Center, Federal University of Pelotas (UFPel), Pelotas 96010-610, Brazil; rafael.delucis@ufpel.edu.br

**Keywords:** pine seed shells, yerba mate, RPUF, sustainable foams, characterization

## Abstract

Pine seed shells and yerba mate are common wastes leftover from the food and beverage industry. This study presents the development of rigid polyurethane foams (RPUFs) filled with pine seed shells and yerba mate at 5, 10 and 15 wt%. The fillers were characterized for chemical properties using bench chemistry analyses, and the RPUFs were investigated in terms of chemical, morphological, mechanical, thermal and colorimetric characteristics. The main results indicated that yerba mate showed good compatibility with the polyurethane system, probably because its available hydroxyl groups reacted with isocyanate groups to form urethane bonds, producing increases in mechanical and thermal properties. However, pine seed shell did not appear to be compatible. Anisotropy increased slightly, as there was an increase in the percentage of reinforcement. The mechanical properties of the yerba mate reinforced foams proved stable, while there was a loss of overall up to ~50% for all mechanical properties in those reinforced with pine seed shell. Thermal properties were improved up to ~40% for the yerba mate reinforced foams, while those reinforced with pine nuts were stable. It was possible to observe a decrease in the glass transition temperature (T_g_) of ~−5 °C for the yerba mate reinforced foams and ~−14 °C for the pine seed shell reinforced ones.

## 1. Introduction

Among the most popular thermosetting plastics, rigid polyurethane foams (RPUFs) are applied in a wide variety of industries, such as building engineering, transport and thermal insulation [1,2]. This versatility is highly attributed to their closed-cell structure, which confers low thermal conductivity, high compressive strength and low water absorption [3]. However, this material has negative environmental impacts due to petroleum consumption and its low biodegradability. In this sense, recent research efforts have been focused on the use of polyols based on vegetable oils [4,5], green chemical additives [6] and fillers based on natural fibres [7,8] or particles [9].

The use of natural fillers from agricultural or forestry wastes has been successfully incorporated in RPUFs, leading to satisfactory performances for maintaining and even increasing the RPUF properties [10]. Increases in several mechanical and thermal properties of RPUFs filled with natural fillers are commonly attributed to the free hydroxyl groups (-OH) on the filler surface, which are available in these lignocellulosic materials and may react with the isocyanate groups (-NCO), generating urethane groups [11]. In addition, the presence of carbonyl and phenolic groups on the filler surface is expected to contribute to a favourable interaction with the polyurethane (PU) matrix, resulting in improvements in several properties. Furthermore, forest and agricultural wastes stand out due to their high availability, low price, high biodegradability and high renewability [12].

In this sense, Członka et al. [13] reported that the insertion of walnut shells in RPUFs changed their cellular structure, resulting in increases in mechanical, thermal and insulating properties. Similarly, Olcay et al. [14] achieved improved mechanical, thermal and sound insulation properties by incorporating RPUFs with artichoke stem fibre wastes. The results reported in both these studies corroborate Delucis et al. [15], who used six forest-based resources (wood, bark, cones, needles, kraft lignin and recycled paper sludge) as fillers in bio-based RPUFs. Based on the mechanical and hygroscopic performances achieved, these authors reported that the wood flour was the most promising filler due to its high holocellulose content. Finally, in the most recent research paper of the group [16], the incorporation of two fruit peels (from banana and bergamot) as fillers in RPUFs yielded increases in cell size and thermal stability. However, there are many residues from the food industry that could be used as fillers in RPUFs, due to their high content of hydroxyls associated with amorphous polysaccharides. The environmental impact of these wastes is a growing concern, particularly in developing countries where inadequate waste management systems can result in pollution and health hazards to both humans and wildlife.

In Brazil and other South American countries, pine seed shells and yerba mate are commonly used for various purposes. Pine seed shells are a popular designation for the seeds of several tree species, including both Pinaceae and Araucariaceae families, and are a popular ingredient for typical dishes from Brazilian gastronomy. Similarly, yerba mate is a traditional South American beverage prepared by infusion, which is widely consumed in countries such as Argentina, Paraguay and Uruguay [17,18]. Although pine seed shells and yerba mate leaves are important goods from the South American culture and cuisine, their consumption also generates wet waste that needs to be managed [19]. In this sense, it is estimated that the annual Brazilian production of yerba mate is greater than 240,000 cubic meters [20], while the production of pine seeds exceeds 30,000 cubic meters per year [21]. This waste generation is even more harmful since both residues are discarded after consumption in the wet form, wherein the high-water content may hinder the recycling process of other residues, such as paper, bottles and cans. In fact, mixing wet food waste can reduce the recyclability by 35% to 50%. Thus, this study aimed to incorporate wastes from yerba mate leaves and pine seed shells in RPUFs.

## 2. Materials and Methods

### 2.1. Acquisition and Characterization of Fillers

Pine seeds (*Araucaria angustifolia* seeds) and yerba mate leaves (*Ilex paraguariensis)* were acquired from local commerce in southern Brazil. Post-consumed yerba mate tea and pine seed shells were oven-dried (at 50 °C until reaching constant mass) and ground in a Wiley mill coupled to a 100-mesh screen (<150 mm). Both these wastes were prepared (Tappi 257 cm-02) using wet chemical analyses, and then the ashes (T211 om-93), ethanol–toluene extractives (Tappi T204 om-97), acid-insoluble (Klason) lignin (Tappi T222 om-98) and holocellulose (remaining mass up to 100%) contents were determined.

### 2.2. Rigid Polyurethane Foam Manufacturing

**Figure 1:**
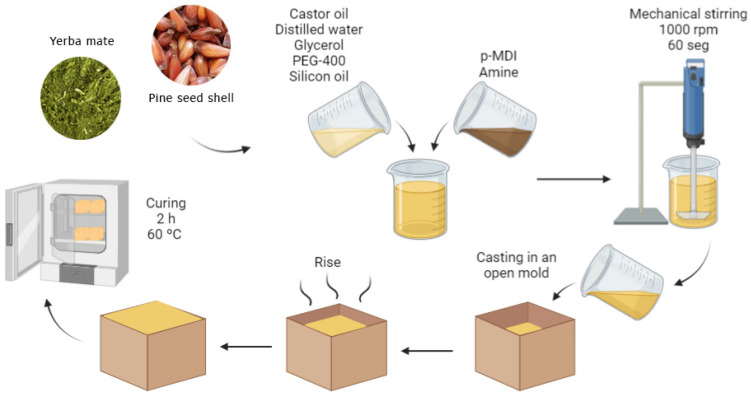
Schematic representation of the RPUF manufacturing.

A bio-based polyol was produced with a simple mixture of castor oil and glycerol at a 3:1 weight ratio. Isotane DM, a polymer methylene diphenyl diisocyanate (p-MDI), was used as an NCO source. Moreover, poly-ethylene glycol (PEG-400), silicon oil and dimethylbenzylamine were used as chain extender, surfactant and catalyst, respectively. For the RPUFs manufacture, castor oil (24 parts/g), distilled water, glycerol (8 parts/g), PEG-400 (3.5 parts/g), silicon oil (1 parts/g) and filler were mechanically mixed at 1000 rpm for 120 s. Thereafter, p-MDI (63 part/g) and amine (0.4 part/g) were added to the other components, which were then mechanically stirred for an extra 60 s, keeping a constant NCO/OH stoichiometric ratio of 1.2. The detailed formulation of the manufactured RPUFs is presented in Table 1, according to others’ research from the group [16,22]. After this process, the liquid reaction mixture was cast in an open mould to freely rise (Figure 1). After its full expansion, the RPUF was cured at 60 °C for 2 h and post-cured at room temperature for two weeks. Filler weight fractions of 5, 10 and 15% were tested.

### 2.3. Morphology and Anisotropy Index

The morphology of the fabricated RPUFs was analysed perpendicular to the rise direction by scanning electron microscopy (SEM) in an MA10 equipment (Zeiss Evo brand) operating at 3 kV. ImageJ software was used to measure average cell width and length using the SEM images. Anisotropy index of the RPUFs was calculated through Equation (1), according to Kirpluks and coworkers [23], where h is cell length, l is the cell width and *n* is number of measured cells.

(1) $$R=\frac{\sum_{i=1}^{n}\frac{h}{l}}{n}$$

### 2.4. Chemical and Thermal Characterization

Fourier-transform infrared spectroscopy (FTIR) was used to identify chemical groups of the fabricated RPUFs and the fillers. IRSpirit equipment (Shimadzu^®^ brand) outfitted with a diamond attenuated total reflection (ATR) accessory was used to record the spectra in the range of 500–4000 cm^−1^ over 64 scans with a resolution of 4 cm^−1^. Moreover, the thermal stability of the RPUFs was evaluated using a TGA-1000 thermogravimetric analyser (TA Instruments, heated from 25 to 800 °C at a heating rate of 20 °C/min under a nitrogen atmosphere). Differential scanning calorimetry (DSC) runs were performed using a Q20 calorimeter (TA Instruments) under a nitrogen atmosphere at a 50 mL/min flow rate from room temperature to 240 °C. The second-order DSC curve, in the range between −25 and 0 °C, was used to determine the glass transition temperature (T_g_) [5,24].

### 2.5. Apparent Density and Compressive Stress

The RPUFs were tested under a compression load parallel to the rise direction using seven prismatic samples (5.0 × 5.0 × 2.5 cm^3^) for each group. The tests were performed in a 23-5D universal testing machine (Emic brand) using a crosshead speed of 2.5 mm/min, and the maximum compressive strength was determined at a 3.3 mm displacement, according to ASTM D1622. Apparent density was determined for the same samples, using an analytical scale (0.001 g resolution) and a digital calliper (0.01 mm resolution).

### 2.6. Colorimetric Patterns of the RPUFs

RPUF’s colour was evaluated using a CR-400 colourimeter (Konica Minolta brand), which reported brightness (L*), green–red (a*) and blue–yellow (b*) coordinates, Chroma (C*) and hue angle (h°). The apparatus was configured to use a light source (illuminant) D65 and a 10° viewing angle, according to the method known as CIELab.

### 2.7. Statistical Analyses

Chemical properties and thermal analysis were performed on one representative specimen. All other data were subjected to ANOVA tests. Whenever the null hypothesis was rejected, Tukey tests were used to compare the means. Before that, homogeneity of variances and data normality were verified using Leven and Shapiro–Wilk tests, respectively. All statistical analyses were implemented at a significance level of 5%.

## 3. Results and Discussion

### 3.1. Chemical Properties

**Figure 2:**
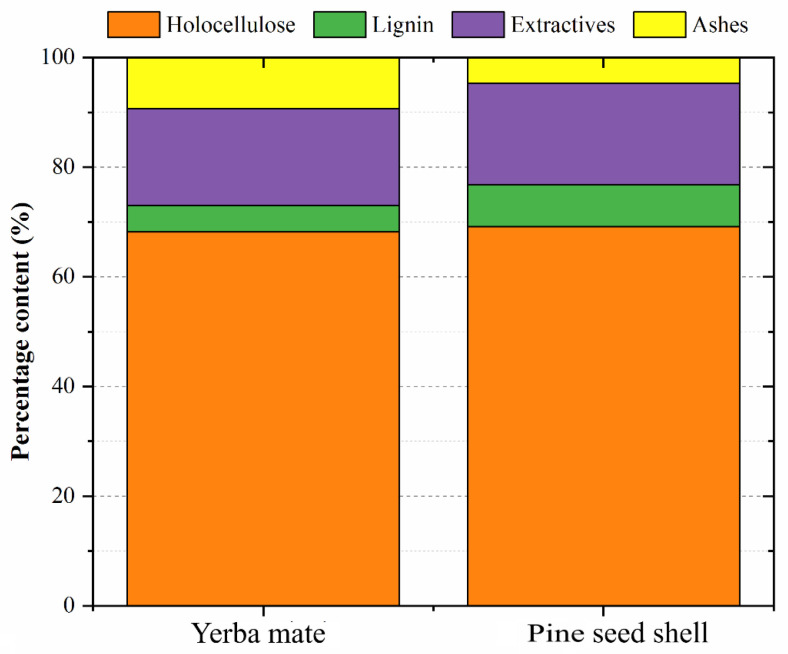
Chemical properties of the pine seed shells and yerba mate leaves.

As expected, both vegetables presented high holocellulose contents (above 60%) [25], indicating that they may have a high host compatibility with the PU system [26]. The pine seed shells presented a slightly smaller holocellulose content when compared to the yerba mate (Figure 2), which presented a higher ash content compared to the former waste. According to the literature [25], among the main hemicelluloses of a typical pine seed shell, the contents of xylans and galactoglucomannans may stand out. These amorphous polysaccharides may have a high OH content free to bond to the PU system. In this sense, Ben and coworkers [27] stated that cellulose, hemicellulose and lignin had 18.52, 3.72 and 3.83 mmol/g hydroxyl content, respectively. Since both residues achieved similar values on holocellulose content (cellulose and hemicellulose), the difference in the hydroxyl content between the two fillers was determined by the lignin content, wherein the pine seed shells fillers had a higher number of these groups.

**Figure 3:**
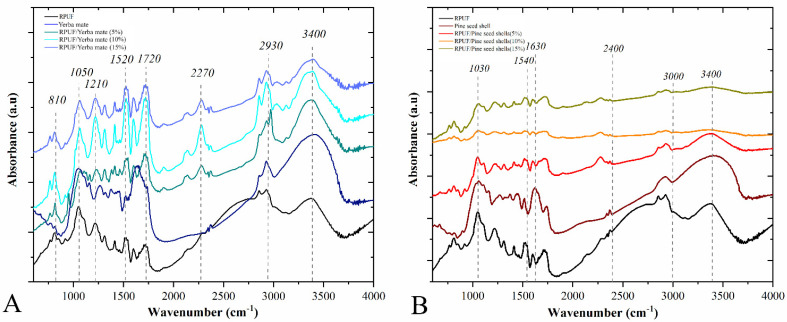
FTIR spectra of the studied RPUFs with yerba mate (**A**) and pine seed shell (**B**), where RPUF is the neat rigid polyurethane foam, and filler type and filler content are the numbers after the bars and between parentheses, respectively.

Compared to the pine seed shell, Figure 3 shows that the yerba mate showed a higher peak at 1720 cm^−1^, which can be attributed to the presence of polyphenols and the -OH bending vibrations from absorbed water molecules [28,29]. Polyphenols are the main active components in yerba mate tea, accounting for approximately 25% of its dry weight [30]. All RPUFs showed similar FTIR spectra, which indirectly indicates chemically similar structures, which are typical of a proper formation of polyurethane chains. These founded chemical groups include C-O-C and C-N urethane linkages (at 900–1200 cm^−1^), N-H amide II (at 1520 cm^−1^), H-C=O urethane linkages (at 1720 cm^−1^), and CH_2_ and CH_3_ groups (at 2850–2970 cm^−1^) [31]. In this sense, the peak in the carbonyl stretching region (1600–1750 cm^−1^) included the free urethane and was lower in the pine seed shell samples. The peak at 2800–3000 cm^−1^ is attributed to symmetric and asymmetric vibrations from methyl (CH_3_) and methylene (CH_2_) groups, as well as C-H groups, associated with amorphous and crystalline polysaccharides [29], allowing for water binding through hydrogen bonds [29,32,33]. These hydrogen bonds were observed by the peak at 3400 cm^−1^, while the peak at 2400 cm^−1^ indicated the presence of NCO groups.

In contrast to the RPUFs incorporated with pine seed shells, the ones filled with yerba mate exhibited an increase in the intensity of the broad and intense peak in the 3200–3500 cm^−1^ region. According to Xu and coworkers [34], this broad peak in PU is associated with the N-H stretching mode, and the absence of a shoulder peak in this region implies the inexistence of unreacted OH groups. In this sense, a small shoulder is observed in the yerba mate samples with 10 and 15% introduction. This behaviour points to a higher number of unreacted contents between the filler and the PU matrix for the yerba mate-filled RPUFs, possibly due to the presence of a high concentration of hydroxyl (-OH) groups on the surface of the yerba mate particles. Therefore, the yerba mate particles contain a variety of functional groups, such as hydroxyls (-OH), carbonyl groups (C=O) and phenolic groups (-C_6_H_4_OH), which may be involved in the interaction with the PU system. Finally, the appearance of a peak at 2270 cm^−1^ related to unreacted NCO groups from the p-MDI was found for the RPUFs filled with 10 and 15 wt% of yerba mate. According to Acosta and coworkers [16], this indicates that these groups were probably trapped in the cellular structure of these RPUFs due to particular nucleation mechanisms conferred by this filler.

### 3.2. Morphology and Anisotropy Index

**Figure 4:**
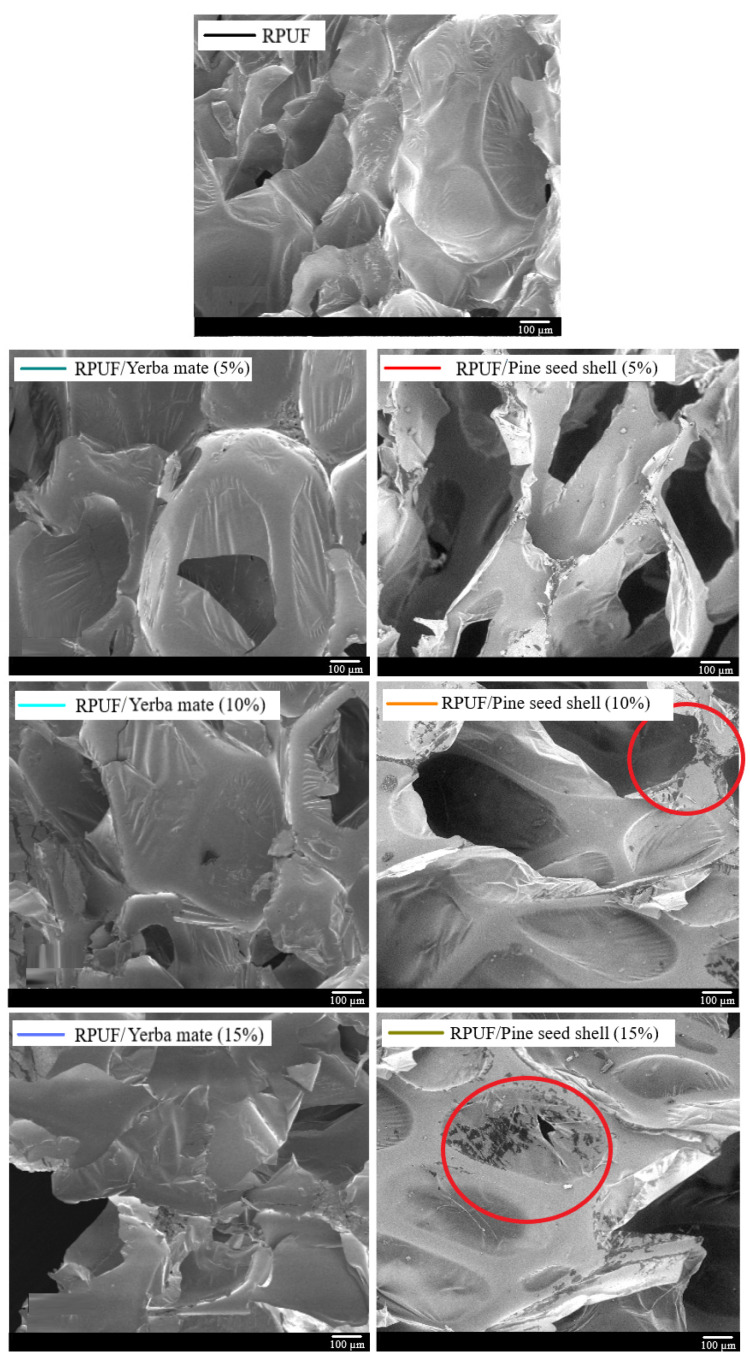
SEM images of the studied RPUFs with yerba mate and pine seed shell, where RPUF is the neat rigid polyurethane foam, and filler type and filler content are the numbers after the bars and between parentheses, respectively.

The hygroscopic, thermal and mechanical properties of an RPUF are highly influenced by its morphology (Table 2). SEM images (Figure 4) clearly showed that the cellular structure of the neat RPUF was mainly composed of closed cells with an elliptical shape oriented in the rise direction, indicating a successful foam formation [23]. As shown in Table 2, this anisotropic behaviour was confirmed by a high anisotropy index of 1.34.

On the other hand, the filled RPUFs presented a high number of open cells, probably due to the disruption of some cells induced by the filler insertion. In fact, it is known that the addition of fillers during the manufacture of RPUFs may cause a change in polymer viscosity, which can negatively impact foam expansion reaction and hinder cell formation and growth, ultimately resulting in an increase in heterogeneity and the emergence of open cells in the filled foams [35].

In general, the insertion of the fillers yielded decreases in cell size, accompanied by unchanged levels of the anisotropy index. The morphological properties of the RPUF incorporated with 5% pine seed shells did not differ from those of the neat RPUF, which indicates a good filler interaction with the PU system in this case. In the study reported by Mosiewicki and coworkers [4], the results showed that decreases in cell size attributed to the incorporation of fillers led to increases in mechanical properties and thermal stability. However, when fillers were incorporated without altering the morphological characteristics of the RPUFs, their properties were similar to those of their respective RPUFs without fillers.

In fact, when the cell size of one RPUF is reduced, its surface area increases, leading to more contact points between the filler and the RPUF matrix. This increased contact may lead to weak bonding between the RPUF matrix and the filler, resulting in low mechanical performance [15]. On the other hand, when fillers are incorporated without altering the cell size of the foam, the contact points between the filler and the foam matrix remain similar to those without fillers. As a result, the bonding between the filler and the RPUF matrix is not weakened, leading to similar mechanical properties [16]. Additionally, the thermal stability of this RPUF is not affected since the surface area of its cells remains unchanged.

The SEM images also indicate that the RPUFs incorporated with yerba mate exhibited a larger cell size than the ones filled with pine seed shells. This corroborates the results obtained by FTIR, and therefore also indicates that there was a better interaction between the yerba mate and its respective RPUF.

### 3.3. Apparent Density and Compressive Stress

**Figure 5:**
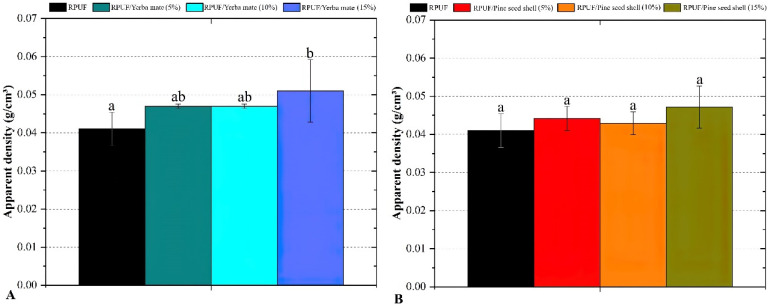
Apparent density of the studied RPUFs with yerba mate (**A**) and pine seed shell (**B**), where RPUF is the neat rigid polyurethane foam, and filler type and filler content are the numbers after the bars and between parentheses, respectively. Different letters represent statistically different means.

As can be seen in Figure 5, the RPUFs’ apparent density is influenced by the amount of filler incorporated, but this relationship is not always linear. According to Acosta and coworkers [16], at low filler concentrations the density may increase as the filler particles occupy space between the RPUF cells, leading to an increase in overall material density. However, at higher filler concentrations, the filler particles can start to agglomerate and form voids in the foam, resulting in a decrease in density. In addition, the incorporation of fillers at high concentrations can also reduce the expandability of the RPUFs, leading to an increase in apparent density. This effect occurs because the presence of the filler particles can hinder the movement and growth of the RPUF cells during the foaming process, leading to a denser and more compact foam structure. Therefore, the optimal filler concentration for achieving the desired balance between RPUF density, cell size/shape and expandability depends on various factors, such as the type of filler, RPUF formulation, processing conditions and intended application [36].

**Figure 6:**
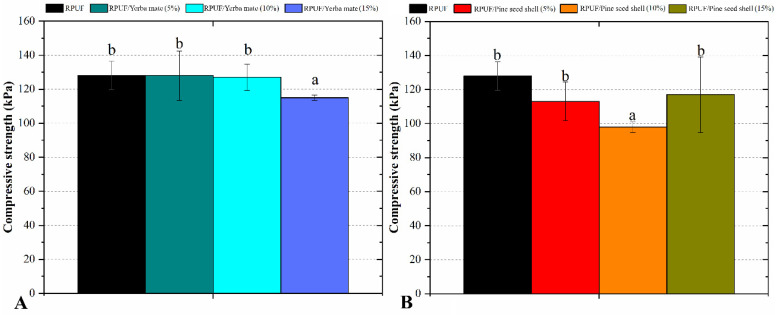
Compressive strength of the studied RPUFs with yerba mate (**A**) and pine seed shell (**B**), where RPUF is the neat rigid polyurethane foam, and filler type and filler content are the numbers after the bars and between parentheses, respectively. Different letters represent statistically different means.

The influences of the pine seed shell and yerba mate wastes on the compressive strength of the RPUFs can be seen in Figure 6. The compression strength values of the RPUFs filled with different mass fractions of pine seed shell and yerba mate wastes remained constant in relation to the same property of the neat RPUF, except for those ones filled with 15% yerba mate and 10% pine seed shell. These latter RPUFs showed significantly lower levels of compression strength compared to the neat RPUF. There are competing and sometimes opposing factors that influence this property, including effects of filler geometry, content and chemistry [15].

**Figure 7:**
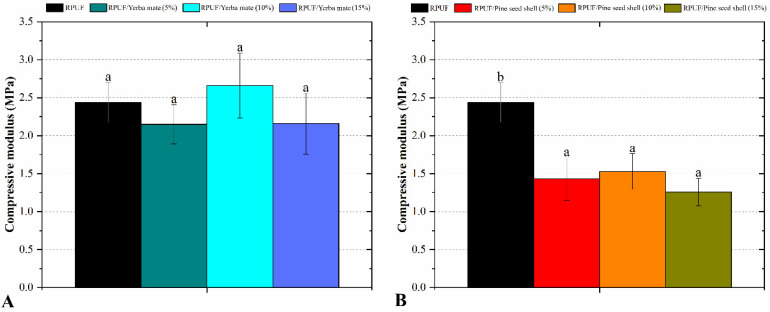
Compressive modulus of the studied RPUFs with yerba mate (**A**) and pine seed shell (**B**), where RPUF is the neat rigid polyurethane foam, and filler type and filler content are the numbers after the bars and between parentheses, respectively. Different letters represent statistically different means.

Regarding the compressive modulus (Figure 7), the introduction of different yerba mate weight fractions did not alter this property. This result again indicates that this filler is compatible with the PU system at a high level. Conversely, all the RPUFs with pine seed shells added presented significant decreases in modulus. In the case of the RPUFs filled with pine seed shells, the decreases in modulus can be attributed to the action of the fillers as stress concentrators, which increases the likelihood of crack formation and propagation within the RPUF structure. This can lead to a reduction in the RPUF modulus, although the presence of these filler particles can also improve the RPUF’s compressive strength by increasing the number of load-bearing points within the RPUF structure. This can offset the negative effects of the stress concentrators and help to explain why the RPUF’s overall strength was maintained.

### 3.4. Thermal Characteristics

**Figure 8:**
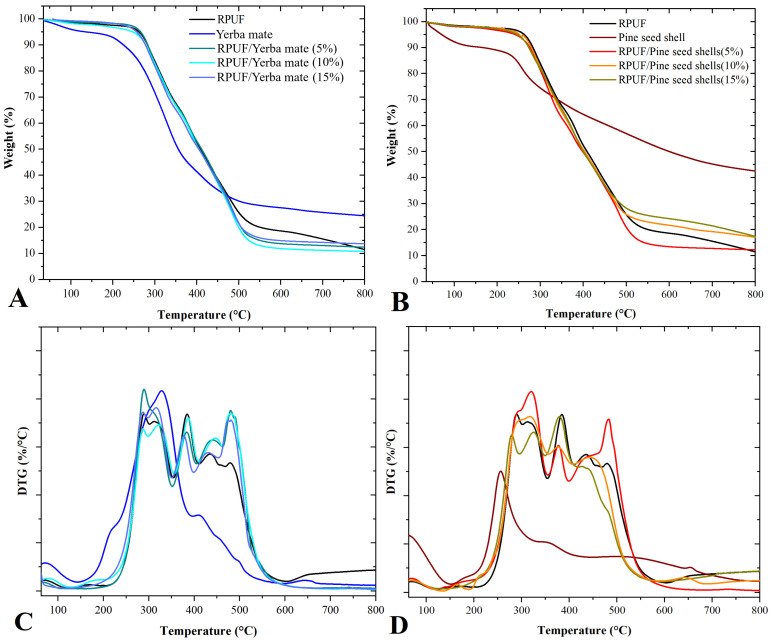
TG (**A**,**B**) and DTG (**C**,**D**) curves of the studied RPUFs with yerba mate and pine seed shell, where RPUF is the neat rigid polyurethane foam, and filler type and filler content are the numbers after the bars and between parentheses, respectively.

Figure 8 shows TG and DTG curves obtained for the studied RPUFs. Characteristic temperatures were defined as T2% (temperature assigned to 2% of weight loss), T5% (temperature assigned to 5% of weight loss) and T50% (temperature assigned to 50% of weight loss) (Table 3).

In all, the shape of the neat RPUF curve remained unchanged after the insertion of the different filler weight fractions, which means that the structural chemical composition of the neat RPUF was almost unchanged. The first important downturn was observed at 240–260 °C. This thermal event was probably due to filler/RPUF crosslinking, since this temperature range is related to the breaking of urethane linkages [16]. In applications such as when building flat roofing sealed with a bituminous roof covering, RPUFs must withstand temperatures up to 250 °C for short periods without showing adverse effects [22]. After that, the T50% results indicate a possible association with the structural decomposition of organic chains, which is governed mainly by the cleavage of urea groups and the degradation of urethane groups, preferentially from side chains around 350 °C [22].

The high residue content observed at temperatures above 700 °C in pine seed shells is associated with the presence of lignin and hemicelluloses, which may become fixed carbon until 600 °C [37]. This may explain the increases in residue content obtained for the RPUFs filled with pine seed shells. This increase in the char content above 700 °C may also lead to decreases in RPUF flammability, since the residue left after thermal degradation can act as a protective layer to the underlying material after successive burning cycles. Therefore, the higher the residue content, the more char will be formed upon thermal degradation, and thus the more fire-resistant the material will be [22].

**Figure 9:**
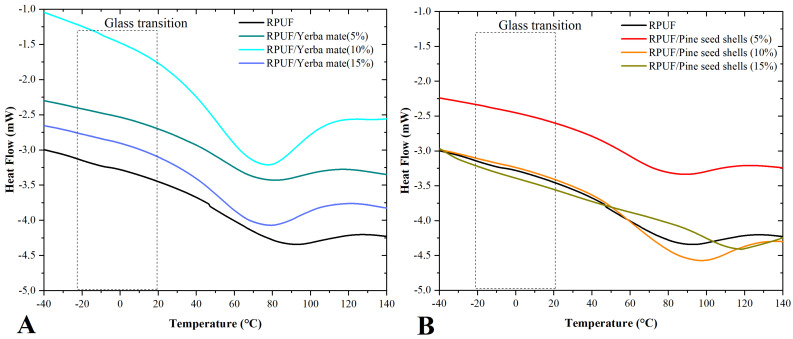
DSC curves of the studied RPUFs with yerba mate (**A**) and pine seed shell (**B**), where RPUF is the neat rigid polyurethane foam, and filler type and filler content are the numbers after the bars and between parentheses, respectively.

Figure 9A,B show DSC curves for both the neat and filled RPUFs. All RPUFs produced DSC curves with somewhat similar shapes, which implies a similar structure of their main macromolecules, as reported for the TG results. The T_g_ of the filled RPUFs shifted to slightly lower values compared with neat RPUF, which may indicate changes in phase separation [38] and damping properties [35]. Although it is hard to find the T_g_ values, the RPUFs filled with yerba mate presented values closer to the neat case, which suggests that filler incorporation decreased the T_g_ values, but that crosslinking bonds had the contrary effect. Additionally, the indicated T_g_ values were in the range found in the literature for RPUFs [24]. The curve shape appears modified within 60–100 °C for RPUFs filled with pine seed shells (Figure 9B). This may be attributed to the release of gas trapped in the cell structure or the volatilisation of some organic extractives, including fatty acids, resin acids, terpenoids and phenolic compounds.

**Figure 10:**
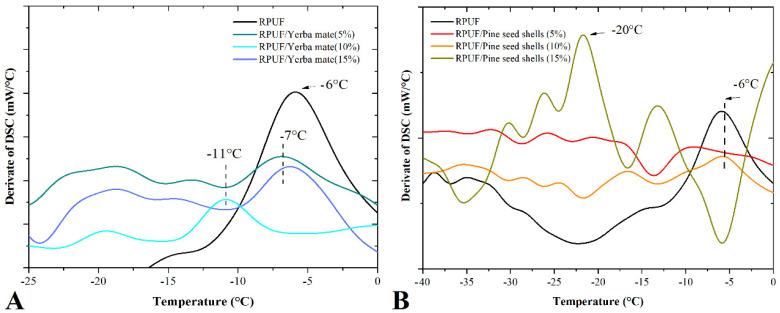
Derived of DSC curves of the studied RPUFs with yerba mate (**A**) and pine seed shell (**B**), where RPUF is the neat rigid polyurethane foam, and filler type and filler content are the numbers after the bars and between parentheses, respectively.

Figure 10 shows the derived curves obtained by DSC. Each prominent peak may represent a significant enthalpy change related to either phase transition or chemical reaction. The shift of the −6 °C peak to a lower temperature (around −10 °C) suggests that the filler may have altered the nucleation and growth behaviour of the RPUF cells, or it may have acted as a nucleating agent, resulting in the formation of smaller cells and a more uniform cell structure [39,40].

Therefore, the addition of the filler in these cases probably changed the way the foam cells formed and grew during the RPUF production process. These fillers may have influenced the size, shape, and distribution of the cells. Additionally, these fillers may have provided sites for the formation of new RPUF cells, resulting in the formation of a more uniform cell structure. A filler that acts as a nucleating agent may also promote the formation of new cells by lowering the energy required for cell formation, which can result in the formation of smaller, more uniform cells.

### 3.5. Colorimetric Patterns

**Figure 11:**
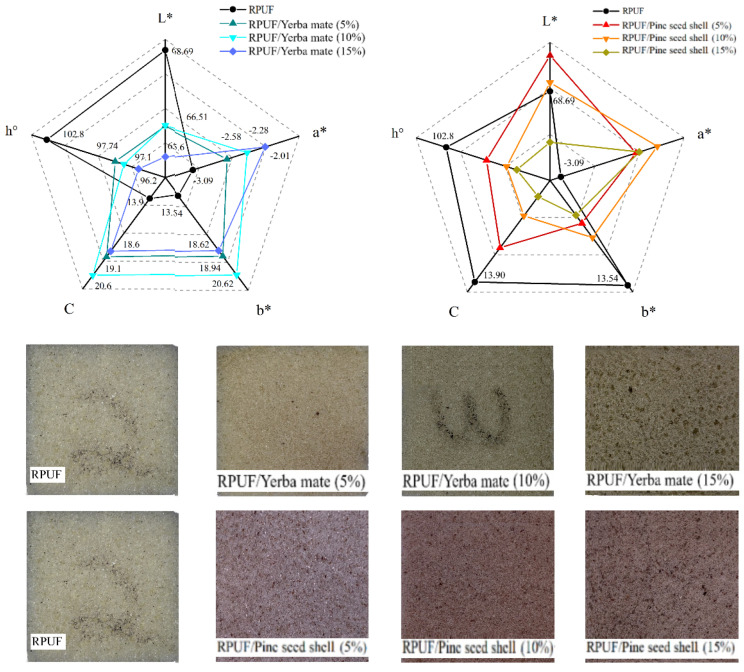
Colorimetric properties and photographs of the studied RPUFs with yerba mate and pine seed shell, where RPUF is the neat rigid polyurethane foam, and filler type and filler content are the numbers after the bars and between parentheses, respectively. Additionally, L* is brightness; a* is green–red coordinate; b* is blue–yellow coordinate; C is chroma; and h° is hue angle.

The photographs shown in Figure 11 indicate that, compared to the neat RPUF, there were significant colour changes attributed to the insertion of both yerba mate and pine seed shells. In general, the filled RPUF became similar to their respective fillers in terms of colour, so the RPUFs filled with yerba mate became green, while the ones filled with pine seed shells acquired brownish shades. In most fruits and vegetables, anthocyanins are among the most known flavonoids that attract consumers because of their colours [41].

All RPUFs incorporated with pine seed shells presented increases in a*, accompanied by decreases in L*, C and h°. On the other hand, the yerba mate being inserted led to decreases in L* and h°, accompanied by increases in a*, b* and C. These results can be explained by the presence of specific compounds in both the pine seed shells and the yerba mate. These complexes may interact with the RPUF matrix, altering their colour properties. For instance, the phenolic compounds (such as polyphenols, quinones and flavonoids) of pine seed shells may be causing the shift in hue from green to red [41,42]. On the other hand, the tannins present in yerba mate may be responsible for the intensification of the red colour, which may explain the increases in a* found for the yerba-mate-filled RPUFs [43]. Regardless of the green–red and yellow–blue coordinates, the significant changes in C found for all the filled RPUFs may represent notable aesthetical changes related to the colour opacity [16].

## 4. Conclusions

RPUF composites incorporating different filler levels (5–15% wt.) of pine seed shells and yerba mate were successfully manufactured. Yerba mate exhibited greater chemical affinity compared to the pine seed shell filler, as confirmed by wet chemical and FTIR results, resulting in more uniform cells found by SEM images. This difference in host compatibility with the RPUF system also significantly affected DSC and mechanical results. The hygroscopic properties, apparent density, compression strength and thermal stability were only slightly influenced by both filler type and content. Although there were evident gains in aesthetic characteristics due to the insertion of both fillers in the RPUFs, the ones incorporated with yerba mate showed mechanical properties similar to neat RPUF, while the RPUFs incorporated with pine seed shells showed losses in the compression modulus.

## Figures and Tables

**Table 1:** Content of the reagents used in the formulation of the foams.

Component	Function	Parts by Weight (php)
Castor oil	Polyol	24.0
Glycerol	Polyol	8.0
PEG-400	Chain extender	3.5
Amine	Catalyst	0.4
Silicon oil	Surfactant	1.0
p-MDI	Polymeric MDI	64.4

**Table 2:** Morphological properties of the studied RPUFs with yerba mate and pine seed shell. where RPUF is the neat rigid polyurethane foam, and filler type and filler content are the numbers after the bars and between parentheses, respectively.

Group	Cell Length (µm)	Cell Width (µm)	Anisotropy Index
RPUF	569 ^(130 cd)^	436 ^(95 c)^	1.34 ^(0.31 ab)^
RPUF/Yerba mate (5%)	659 ^(266 de)^	565 ^(202 d)^	1.20 ^(0.33 ab)^
RPUF/Yerba mate (10%)	652 ^(164 de)^	428 ^(101 bc)^	1.54 ^(0.31 c)^
RPUF/Yerba mate (15%)	723 ^(271 e)^	572 ^(181 d)^	1.37 ^(0.33 bc)^
RPUF/Pine seed shell (5%)	508 ^(113 bc)^	422 ^(132 bc)^	1.35 ^(0.56 ab)^
RPUF/Pine seed shell (10%)	397 ^(105 ab)^	271 ^(66 a)^	1.48 ^(0.30 ab)^
RPUF/Pine seed shell (15%)	323 ^(83 a)^	319 ^(66 ab)^	1.07 ^(0.36 a)^

Where: RPUF is the neat rigid polyurethane foam, and filler type and filler content are the numbers after the bars and between parentheses, respectively. Different letters represent statistically different means.

**Table 3:** Main thermal events evaluated by TG analysis of the studied RPUFs with yerba mate and pine seed shell, where RPUF is the neat rigid polyurethane foam, and filler type and filler content are the numbers after the bars and between parentheses, respectively.

Group	T_2%_	T_5%_	T_50%_	Residue at 800 °C (%)
RPUF	160	262	404	12
Yerba mate	62	135	355	25
RPUF/Yerba mate (5%)	218	266	409	12
RPUF/Yerba mate (10%)	119	247	405	11
RPUF/Yerba mate (15%)	215	256	404	14
Pine seed shell	45	70	599	45
RPUF/Pine seed shell (5%)	103	241	398	12
RPUF/Pine seed shell (10%)	157	251	401	19
RPUF/Pine seed shell (15%)	121	252	402	21

## Data Availability

The study did not report any data.
